# Dementia Education and Training for In-Patient Health Care Support Workers in Acute Care Contexts: A Mixed-Methods Pilot Evaluation

**DOI:** 10.3390/ijerph22060860

**Published:** 2025-05-30

**Authors:** Leah Macaden, Kevin Muirhead, Juliet MacArthur, Siobhan Blair

**Affiliations:** 1 Nursing Studies, School of Health in Social Science, University of Edinburgh, Edinburgh EH8 9AG, UK; 2 School of Health and Life Sciences, University of the West of Scotland, Paisley PA1 2BE, UK; kevin.muirhead@uws.ac.uk; 3 School of Nursing and Midwifery, Queen’s University, Belfast NHS Lothian, Edinburgh EH3 9DN, UK; juliet.macarthur@nhs.scot; 4 NHS Lothian, Edinburgh EH3 9DN, UK; siobhann.blair@nhs.scot

**Keywords:** dementia education, training, acute care, mixed method, health care support workers

## Abstract

Aim: To understand dementia care knowledge, attitudes, and confidence among acute-care support staff following a dementia education intervention titled Dementia Workforce Excellence in Acute Care. Design: A convergent parallel mixed-methods pilot study. Methods: Data were collected from 30 participants using an online survey and three individual interviews between January and March 2024. Survey data were analysed using descriptive statistics, and a thematic analysis underpinned by Kirkpatrick’s framework was used to analyse the qualitative data from interviews. Results: The online survey established good levels of dementia knowledge, attitudes, and confidence among support staff with enhanced attitudes among staff who completed the training. Analysis of interviews resulted in three themes: dementia in the acute care setting; motivation for learning; and evaluation of the intervention on four levels [satisfaction, learning gains, behaviours, and results]. Conclusion: Findings suggest that the dementia education intervention used in this study is a comprehensive dementia training resource that promotes person-centred and compassionate dementia care across all stages of the dementia journey. Dementia is a public health priority with workforce education identified as a key response for capacity building. This pilot evaluation offers insight and new learning on the pedagogical approaches that are inclusive of peer-supported reflective learning in small groups that remain untapped for dementia workforce development. Dementia inclusive and enabling environments with a knowledgeable and skilled workforce are crucial to mitigate stigma and discrimination. This can be best achieved by raising awareness through targeted staff education and training to make hospital environments more dementia inclusive. Patient or Public Contributions: Dementia care scenarios used in this study were co-designed by experts with lived experience of dementia. Additionally, these experts along with family carers of people living with dementia were involved in the delivery of the training where appropriate.

## 1. Background

The global prevalence of people living with dementia is 57 million, with 10 million new cases anticipated annually [1]. Within the UK, seven percent of older adults aged over 65 are estimated to be living with dementia, and future prevalence is predicted to resemble global trends due to population projections and risk with ageing of developing dementia [2,3]. Despite evidence suggesting a decline in dementia-specific incidence in some high-income countries, dementia continues to pose multidimensional challenges with significant implications for affected individuals, their families, social policy, and national economies [4]. Dementia costs the UK economy £ 42 billion per year, which poses a significant challenge for the NHS since an estimated 25% of acute care beds are occupied by people with dementia who are hospitalised, more than the general population, and who experience longer average lengths of stay [5,6]. Acute hospital admissions pose exceptional challenges for people with dementia experiencing acute illness or injury within unfamiliar and disruptive environments, which can contribute to an increased risk of adverse events, including delirium, falls, and inappropriate pain management [7,8]. This contributes to a downward cascade of interacting problems exacerbating cognitive and functional decline and resulting in increased dependency [8] contributing to bottlenecks with bed availability and suboptimal care transitions to care homes and care at home.

Acute hospital wards are often understaffed with varying ratios of registered to unregistered staff [8]. Acute care support staff [ACSS] are unregistered front-line staff who care for people with dementia and can have a powerful influence on care quality [9]. However, environmental factors and organisational pressures limit ACSS’s ability to provide person-centred dementia care [10]. Disruptive patient behaviours also impact staff routines, and, when misunderstood, they can be a source of stress and frustration for both staff and patients. Negative attitudes can contribute to belief systems that behavioural and functional decline is inevitable, which influence staff’s knowledge, confidence, and ability to differentiate between acute and chronic states, e.g., dementia versus delirium [8]. Findings from a systematic review [11] suggested that nurses’ dementia knowledge and attitudes improved after the implementation of training programmes. A cross-sectional study in Vietnam [12] reported deficits in knowledge, nurses’ attitudes, and a lack of confidence, especially in caring for patients with dementia who could not communicate verbally. This suggests that there is a need for dementia education and training that is aimed towards enhancing staff knowledge influencing attitudes and building their confidence whilst recognising the emotional and environmental challenges experienced by ACSS caring for people with dementia in acute hospital settings [7,13].

In Scotland, a series of dementia strategies [14,15,16,17] and the implementation of the Promoting Excellence Framework [18]—Scotland’s first dementia workforce development framework—have been integral to the national work on dementia by setting out the knowledge and skills that all health and social care staff should achieve in their roles to support people with dementia, their families, and carers. The framework puts great emphasis on human rights and integrates the dementia journey at four practice levels: informed, skilled, enhanced, and expert. Each level corresponds to the frequency and intensity with which practitioners engage with people affected by dementia within their unique practice settings.

Despite dementia education for the health and social care workforce being embedded into national policy and guidance, the optimal way of developing the workforce remains unclear [19]. There are currently a large number of learning resources available for dementia training; however, several are geared towards episodic care, e.g., understanding stress and distress in dementia, and do not consider the dementia journey or focus on the person behind the condition. Existing training programmes may be suboptimal for ACSS, who will meet people with dementia and their families anywhere along their dementia journey and require education that can convey the complexities and progressive nature of the condition. Furthermore, the resources available to acute hospital staff often involve asynchronous online learning due to challenges with providing time for in-person training driven often by staffing shortages. Asynchronous learning does not encompass interactive participation in real time, which is recommended as a core component of effective dementia education and training [20]. Previous research has demonstrated that impactful dementia training programmes include those delivered face to face with a blend of online learning [21].

There is a dearth of current evidence for blended dementia education for acute care practitioners. Toubøl et al. [22] described a blended programme for acute hospital staff with the aim of improving basic competencies among the entire workforce in a general hospital. The training resulted in improvements in dementia care knowledge, competence, and attitudes across the acute care workforce. In addition, from what is known, there is a lack of formal evaluation of the dementia training resources available to ACSS in Scotland. Dementia Workforce Excellence in Acute Care [DWEAC] is a comprehensive dementia training programme that blends online and face-to-face learning with content mapped along the stages of the dementia journey from pre-diagnosis to end of life with emphasis on person- and relationship-centred care using reflective and interactive pedagogical methods.

### Aim

The aim of this pilot study was to evaluate DWEAC as an educational intervention to enhance dementia care knowledge, attitudes, and confidence among acute care support staff in a large health board in Scotland.

## 2. Methods

### 2.1. Design

A mixed-methods evaluation was used using a convergent parallel mixed-methods approach combining online survey response data with qualitative data from interviews.

### 2.2. The Intervention

DWEAC integrates the philosophy of care within the Standards of Dementia Care in Scotland [23] that are underpinned by the National Dementia Strategies. Programme development was informed by the Promoting Excellence Framework and aligned to skilled level, which outlines the knowledge and skills required by ACSS since they have direct and/or substantial contact with people living with dementia. DWEAC adopts the “journey approach” to dementia education, which the project lead (LM) previously introduced in Being Dementia Smart [24] and Dementia Education for Workforce Excellence [25].

The blended approach in DWEAC allows participants to learn with flexibility from three online workbooks alongside real-time participation in a classroom environment. Learning content is mapped along the stages of the dementia journey with an emphasis on the relationship between retained/enhanced personhood and wellbeing [person-centred care] [26,27] and the dynamic yet crucial interplay between people with dementia, their families, and formal carers [relationship-centred care] [28]. The key narrative is based on “Barbara’s Story”, a filmed ethnodrama that demonstrates the complexities of living with dementia and experiencing acute care [19]. Learning content is supported by active learning strategies and opportunities for personal reflections. Synchronous sessions are delivered over full days and facilitated by experts including professionals and people with lived experience of dementia and dementia caregiving. The sessions uphold the philosophy of person-centred dementia care and are regularly interspersed with opportunities for group interaction, reflection, and feedback [see Table 1 for details on the indicative content].

The research questions [RQ] were as follows:

1. What are the levels of dementia knowledge, attitudes, and confidence among acute care support staff?
2. Is DWEAC an effective educational intervention to improve dementia knowledge, attitudes, confidence, and person-centred care practices?
3. How do acute care support staff perceive the content and quality of DWEAC?
4. How does DWEAC need to be tailored further to ensure relevance for acute care support staff?

### 2.3. Sampling and Recruitment

A member of the research team [SB] served as the gatekeeper and liaised with senior charge nurses from five clinical areas [stroke medicine, medicine of the elderly, orthopaedics, general medicine, and Department of Clinical Neuroscience] across three acute hospital sites. SB negotiated support for the study with these departments to disseminate project information and create awareness of the study. All ACSS [*n* = 220] from the five clinical areas [including DWEAC participants] were emailed a link to an online questionnaire by a member of the research team [SB], the purpose of which was to establish levels of dementia knowledge, attitudes, and confidence among ACSS [RQ1]. Ten percent of the ACSS [*n* = 20] from the above areas were recruited to attend DWEAC training in person over three days with access given to online workbooks ahead of the training.

All DWEAC participants were informed about the evaluation during the training and invited to participate in interviews. Those who expressed an interest provided their contact details to a member of the research team [SB]. Another member of the research team [KM] subsequently emailed those interested with participant information and a link to provide online consent. Interviews were scheduled for consenting participants.

There were challenges with recruiting to both components of the study despite a robust recruitment strategy via the gatekeeper [SB] in her role as the dementia nurse consultant and two email reminders with the survey link to all ACSS in the five areas that were identified for this study. Two separate email reminders were also sent to the ten DWEAC participants who expressed interest to participate in the interviews following the training. This is thought to be due to the current staffing pressures in acute care within the National Health Service [NHS]. Given that the study was funded for only 6 months and the ethics approval effectively took three months, no further strategies could be explored to enhance recruitment.

### 2.4. Data Collection

Quantitative data were collected via the Jisc Online Survey Platform [version 3] [29] approximately one month after DWEAC training. Consent was gained via the survey platform. The time points for data collection aimed to establish the impact of the training whilst mitigating the potential for recall bias.

Single interviews [45–60 min each] with each participant were conducted virtually via Microsoft Teams within two months of participation in DWEAC. Consent for interviews was obtained electronically using the same survey platform via a separate link.

The online survey utilised three measures, the Knowledge in Dementia [KIDE] scale [30], the Confidence in Dementia [CODE] scale [30], and the Dementia Attitude Scale [DAS] [31], to measure dementia knowledge, confidence, and attitudes, respectively. Knowledge items were 16 statements about dementia/dementia care with binary true/false response options. Attitude items were 20 statements about caring for people with dementia on a 7-point Likert scale [strongly disagree to strongly agree]. Confidence was measured using a similar approach with nine items on a 5-point Likert Scale [not at all confident to extremely confident].

Interviews were semi-structured using an interview guide [Supplementary Material] and were inclusive of three reflective-themed dementia care scenarios around person-centred dementia care developed by dementia care experts with professional and lived experience of dementia and dementia caregiving. Participants were presented with a scenario [examples of these scenarios are presented in Tables 9–11] at the interview. Discussions were focused on how they would have cared for the person in the scenario admitted to their ward prior to their training versus following their training. Participants were asked to consider a scenario and then share their approaches to dementia care before and after taking part in DWEAC.

### 2.5. Data Analysis

Survey data were analysed in Microsoft Excel. Demographic data were reported as frequencies and percentages. Items on KIDE were scored as being correct [score = 1] or incorrect [score = 0] with data reported using descriptive statistics for all participants and those who did and those who did not attend DWEAC. Chi-squared tests were used to compare scores between participants who did and did not attend the training. CODE response data were reported using descriptive statistics and displayed graphically for all participants. Median values with interquartile range [IQR] were reported to demonstrate differences between those who did and those who did not attend DWEAC. Items on DAS were classified as being positive (*n* = 14) or negative (*n* = 6) statements about caring for people with dementia. Positive and negative items were reported using descriptive statistics and displayed graphically for all participants. Median values with IQR were presented to demonstrate differences between those who did and did not attend DWEAC. Negative items [2, 5, 8, 9, 16, and 17 from the original scale] were reverse scored and then combined with positive items to achieve total median scores and IQR. Interview data were subject to reflexive thematic analysis [32]. Analysis comprised six phases: (1) interview recordings were transcribed verbatim and reviewed multiple times by a member of the research team [KM]; (2) data were coded by one member of the research team [KM] using NVivo; (3) initial codes were clustered into provisional themes through a process of development, revision, and refinement in consultation with LM; (4) themes were reviewed in relation to codes and the data set to consider alternative options for pattern development; (5) a detailed thematic framework was produced; and (6) an analytic narrative was created with data extracts. The analytical process was initially inductive; however, the thematic framework in development resembled Kirkpatrick’s four-level model of training evaluation [33], which resulted in codes being reviewed and applied deductively into themes reflecting learner satisfaction, learning gains, behavioural change, and results due to the training. Qualitative and quantitative findings were reviewed and converged with equal priority. Areas of convergence, divergence, or expansion across both data sources were identified and reported narratively and in summary tables.

### 2.6. Ethical Approval

Ethical approval was gained from REDACTED, NHS management approval [including site-specific and information governance/IT security approval] was gained from REDACTED. Ethical and management approval was supported by Research Governance at REDACTED. Final approvals were subject to organisational agreements between the sponsor REDACTED, and a sponsor-approved Data Protection Impact Assessment.

## 3. Results

### 3.1. Quantitative Findings

#### 3.1.1. Participant Demographics

Participants [*n* = 30] were clinical support workers [46.7%], health care assistants [16.7%], occupational therapy [OT] assistants [10%], and physiotherapy [PT] assistants [6.7%]. Other professionals included a PT and OT assistant, a speech and language therapy assistant, a radiology departmental assistant, an activities coordinator, and two assistant practitioners. Most participants worked in general medicine [36.7%] or medicine of the elderly [MOE] [36.7%] with the remaining working in the Department of Clinical Neuroscience, orthopaedics, and stroke medicine. There were four survey respondents [13.3%] who attended DWEAC training [Table 2].

#### 3.1.2. Experience of Dementia Care and Motivation for Learning

**Figure 1:**
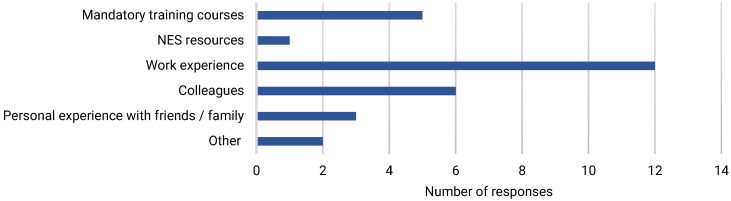
Main source of knowledge on dementia.

**Figure 2:**
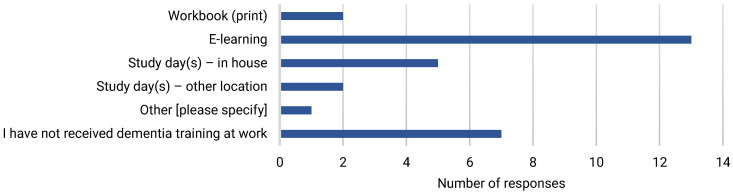
Form of most recent dementia training.

**Figure 3:**
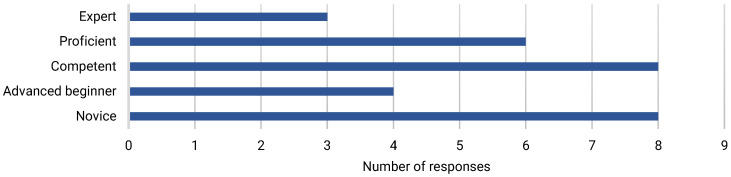
Self-perceived competence in dementia care.

Most survey respondents [60%] had more than five years’ dementia care experience, and half had more than five years’ experience working in acute care [Table 2]. Sources of learning about dementia included work experience [40%], learning from colleagues [20%], and through personal experience of informal dementia care [10%] [Figure 1]. E-learning was the most common form of formal dementia training accessed [Figure 2]. Most respondents [56.7%] considered themselves to be at least competent in dementia care [Figure 3], and most [63.3%] believed that they had the knowledge and skills to provide care for people with dementia at work. However, less than half [43.3%] felt that their dementia training needs were currently being met.

#### 3.1.3. Dementia Knowledge

High levels of dementia knowledge among all ACSS were reflected in an average percentage score of 82.8% across all KIDE items. Items 1, 3, and 4 had the lowest percentage scores [≤60%], suggesting knowledge gaps in aspects of dementia aetiology and disease progression. There was no significant difference in the total mean percentage score for those who did [81.3%] and those who did not attend DWEAC training [83.0%], *p* = 0.729. However, scores from DWEAC participants trended towards greater knowledge for items 4, 6, 9, 10, 14, and 16, suggesting that the training may have helped to address some of the knowledge gaps identified in the wider group and resulted in a greater understanding of pain management for people with dementia in acute care settings [Table 3].

#### 3.1.4. Dementia Care Confidence

**Figure 4:**
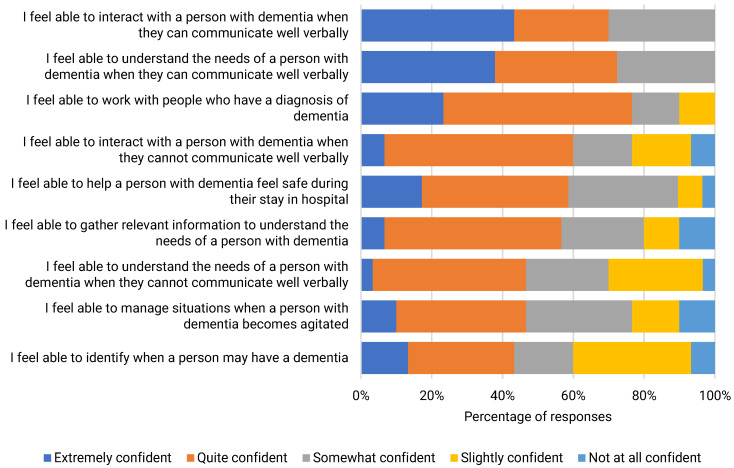
Dementia care confidence among ACSS.

There were good levels of dementia care confidence among ACSS. Confidence to work and interact with people with dementia was highest when patients were able to communicate well verbally and lowest when patients demonstrated agitated behaviours. ACSS also had less confidence identifying when a person may have dementia [Figure 4]. DWEAC participants had slightly higher levels of confidence in their ability to identify when a person may have dementia, manage situations when a person with dementia becomes agitated, and understand the needs of a person with dementia whether or not they could communicate well verbally [Table 4].

#### 3.1.5. Dementia Care Attitudes

**Figure 5:**
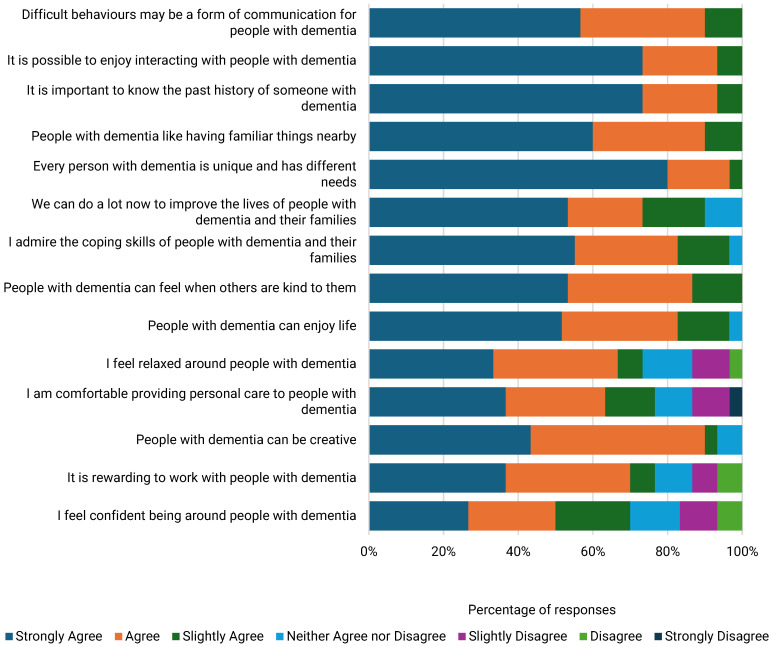
Positive items reflecting dementia care attitudes among ACSS.

**Figure 6:**
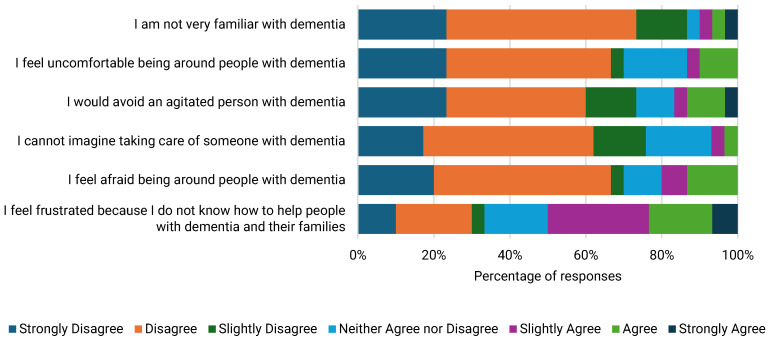
Negative items reflecting dementia care attitudes among ACSS.

ACSS demonstrated having positive dementia care attitudes based on responses to almost all DAS items. However, a notable exception was feelings of frustration due to a lack of knowledge and skills on how to help people with dementia [Figure 5 and Figure 6]. Those who attended DWEAC demonstrated greater awareness of ways to help people with dementia and were more likely to recognise their abilities. DWEAC participants also demonstrated greater optimism about dementia, comfort being around people with dementia, and satisfaction when working with people with dementia [Table 5].

### 3.2. Qualitative Findings

#### 3.2.1. Participants

Three ACSS who completed DWEAC training participated in interviews. Participants were clinical support workers from general medicine [*n* = 2] and a PT assistant from MOE. It is not known whether interview participants completed the online survey since this was completed anonymously.

#### 3.2.2. Themes

Three themes developed from the analysis: (1) dementia in the acute care setting; (2) motivation for learning with DWEAC; and (3) four-level evaluation of DWEAC. The third theme comprised four subthemes: (1) satisfaction; (2) learning gains; (3) behaviours; and (4) results.

#### 3.2.3. Dementia Care Practice in the Acute Care Setting

Participants felt that they were well placed to provide good quality dementia care to patients but were constrained by the demands in an acute care environment as demonstrated by data in Table 6.

#### 3.2.4. Motivation for Attending DWEAC

Participants often acquired skills for dementia care informally “on the job” [P3]. The need for formal education to consolidate practice was intensified by the significant change in patient demographics in acute care settings.

*“Also, for the job that I was doing because where I am now, albeit that I’m in a [acute] ward, our ward’s fast becoming, on a daily basis, just an MOE ward”.*

P2.

Opportunities for dementia education at work were not always obvious and, where available, were perceived to be limited with inadequate content.

*“I did a one-day thing, in fact it wasn’t even a whole day … it was within the [Hospital], but it wasn’t very detailed”.*

P1.

The main motivating factor for attending DWEAC was the development of knowledge and skills to care well for people with dementia.

*“Rather than just touching on the surface, I wanted to get more in-depth information about different types and, you know, and just to also improve my skills when dealing, my knowledge when dealing with these patients, you know, and hopefully being able to help them, you know, and I wouldn’t say understand but, you know, like to be able to like communicate better with them and have a better understanding of what they’re going through”.*

P2.

Some participants were motivated by personal interest and a desire to improve informal dementia care for their own family members.

*“My mum’s got dementia so … trying to deal with my mum like better, you know, and maybe understanding what she’s going through … trying to take away bits and pieces”.*

P2.

### 3.3. A Four-Level Evaluation of DWEAC

#### 3.3.1. Satisfaction

The participants anticipated that DWEAC would be similar to existing dementia training resources available and did not expect the comprehensive learning and interactive opportunities with involvement of people with lived experience of dementia. All three participants expressed their satisfaction with DWEAC, which was perceived to be “very positive” [P1], “great” [P2], and “really well done” [P3], and found the training to be both enlightening and thought-provoking. Additionally, participants perceived that their learning experience was enhanced from content delivered by experts with professional experience of dementia who were considered to be accessible and approachable.

The face-to-face training was considered to be well-paced over three days with time for participants to absorb and reflect on the learning content.

Involving people with lived experience of dementia inspired participants to see the person behind dementia and resulted in greater depth of emotional engagement. Although participants felt underprepared for the emotional intensity of the learning experience, they expressed high levels of satisfaction on the content, mode of delivery, and quality of training as demonstrated by participant quotes in the table below [Table 7]:

#### 3.3.2. Learning Gains

Learning gains following DWEAC are illustrated with representative participant quotes in Table 8.

#### 3.3.3. Behaviours

Prior to DWEAC training, participants reported making assumptions about people with dementia and did not always see the person behind the illness. Interactions with people with dementia could be based on instinct rather than robust clinical knowledge despite some awareness of policy and procedures for dementia care in the acute setting including existing pathways to get to know the patient better.

*“I would have just been going on my instinct and just tapping into who’s been looking after him and reading about what matters to him in his wee folder”.*

P2.

Participants also demonstrated awareness of the relationship between patients’ unmet needs and stressed/distressed behaviours. However, support for clinical decisions could be sought from senior colleagues despite opportunities for ACSS to implement psychosocial interventions independently.

*“My first point of contact would have been going to my nurse in charge or the nurse who’s looking after that patient”.*

P2.

Following DWEAC training, participants described even greater awareness of the physical, emotional, and unmet needs of people with dementia and reported being more attuned to the possibility of alternative pathologies when interpreting behaviours. There was also evidence of improved communication, compassionate care, and attention to personhood.

*“I would deal with her a bit better and with more understanding … more empathy”.*

P3.

Table 9, Table 10 and Table 11 illustrate the dementia care scenarios presented to participants. Quotations represent participants’ perceived behaviours before and after attending DWEAC training.

#### 3.3.4. Results

Learning with DWEAC appeared to have had a positive impact on participants’ dementia care practice.

*“I’d been put on a different base and the relative asked if I could go and work on the other base because her mum seemed much, more settled when I was around … what an honour that was”.*

P1.

Following the training, participants reported having confidence to share new knowledge with colleagues including developing resources such as information booklets and displays to create a ripple effect from learning with potential to improve dementia care quality

*“For you giving me the opportunity to be on this course, I’ve now passed a lot of that information on, on the ward, since I’ve been back on the ward”.*

P2.

*“I’ve already started putting a booklet together for the setting”.*

P1.

*“I like a display … I think visual stuff I’m better at … it encourages conversations…people have been asking me, coming up to me and asking me questions”*.

P2.

*“When I finished the course … my Band 7 had asked me would I do a presentation to the team … I did a presentation for the team a couple of weeks ago which seemed to go down really quite well”.*

P3.

Participants considered that they were well-placed to train colleagues following DWEAC with the caveat being that training sessions should not involve large groups.

*“It would depend on how many were there … I don’t mind doing it for kind of the smaller groups … bigger groups, that would maybe kind of phase me but, you know, smaller groups… I wouldn’t be phased by it”.*

P3.

### 3.4. Mixed Methods Interpretation [Please See Supplementary Files S1–S3]

#### 3.4.1. The Need for Dementia Education in Acute Care

Qualitative findings suggested that the increasing dementia prevalence in acute care settings has intensified the need for staff to be well educated about dementia. Despite this, quantitative findings highlighted that not all ACSS are prepared with the knowledge and skills to care well for people with dementia. Quantitative findings confirmed that most ACSS learn about dementia through work experience, with qualitative findings suggesting that ACSS are well placed to provide person-centred dementia care despite workforce challenges. Quantitative findings revealed that some ACSS provide informal dementia care to family members, which qualitative findings expanded on, suggesting support for informal dementia care as an additional motivating factor for participation in DWEAC. Quantitative findings suggested e-learning to be the most common form of dementia training available to ACSS. Qualitative findings expanded on this, suggesting that ACSS may lack awareness of the opportunities available for dementia training at work.

#### 3.4.2. Dementia Care Confidence and Competence Before DWEAC

Quantitative findings suggested that ACSS have high levels of dementia knowledge. Qualitative findings expanded on this, highlighting participants’ pre-training awareness of person- and relationship-centred care, unmet needs of people with dementia, and local dementia policy and procedure. However, qualitative findings also suggested that ACSS could rely on instinct rather than robust clinical knowledge when caring for people with dementia. Good levels of dementia care confidence and positive attitudes towards people with dementia were established from the quantitative findings. This diverged somewhat from the qualitative findings, where participants reported making assumptions about people with dementia and not always seeing the person behind the illness. There was also some evidence that participants lacked confidence to implement psychosocial interventions independently.

#### 3.4.3. Dementia Care Confidence and Competence After DWEAC

Quantitative findings suggested that DWEAC may have addressed some dementia knowledge gaps. Qualitative findings expanded on this, suggesting that DWEAC participants developed knowledge on person- and relationship-centred dementia care, different forms of dementia, and a greater awareness of the physical, emotional, and unmet needs of people with dementia. Participants also developed proficiency in their ability to care for people with dementia through enhanced communication skills and being more attuned to the possibility of alternative pathologies [delirium for example] when interpreting behaviours. In general, the training resulted in a broader understanding of dementia for application in both professional and informal care settings. The quantitative findings suggested that DWEAC may have resulted in greater optimism about dementia, comfort being around people with dementia, and satisfaction when working with people with dementia. This was expanded on in qualitative findings, which suggested that dementia care confidence had increased to the extent that participants were more conscious of the assessments and decisions they made in practice and were subsequently able to share new knowledge with colleagues. Quantitative findings suggested that DWEAC may have helped to improve dementia care attitudes. This was further expanded on during the interviews, which suggested that participants paid greater attention to personhood and cared for people with dementia with greater compassion and empathy since participating in DWEAC. Details on the mixed methods integration is provided as Supplementary Material.

## 4. Discussion

Findings from this pilot evaluation suggest that DWEAC may enhance existing levels of dementia care knowledge, attitudes, and confidence among ACSS. Consistent with existing evidence, this evaluation highlights the increasing number of people with dementia in acute hospital settings and the need for comprehensive dementia training programmes to develop the workforce. Increased levels of contact with people with dementia provide ACSS with informal learning opportunities but lack the necessary integration of theory [34] to better understand the “progression/trajectory of the illness and person behind the illness”. ACSS appreciate the need to learn from both perspectives [7] and welcomed access to DWEAC to bridge knowledge and skills gaps. The blended approach complemented self-paced learning with interactive and case-based learning in real time, which previous dementia education research has established to be effective pedagogical practices for general hospital staff [35].

DWEAC was designed by dementia care experts and delivered by experienced facilitators with learning pitched at the right level for ease of understanding. Knowledge of different forms of dementia had particular relevance to ACSS who will provide care for a variety of inpatients with various dementia subtypes [36]. Broader knowledge of dementia and dementia caregiving was gained from experts with lived experience, which is known to benefit acute care staff attending dementia training [37]. Realities around disease progression and its associated complexities were further developed using an authentic clinical case, Barbara’s story, which powerfully communicated the human experience by allowing learners to immerse themselves in the life of someone living with dementia [19]. The focus on person-centred dementia care throughout DWEAC reinforced the importance of seeing the person behind the condition, which is inextricably linked to dementia-specific knowledge, interpersonal skills, and empathetic abilities [38]. The relationship-centred care aspects promoted an inclusive approach to dementia care, putting the person with dementia at the centre of care whilst recognising the involvement and needs of formal and informal carers [39,40,41].

Immense workloads and time pressures in acute care settings can limit opportunities for patient- and relationship-centred dementia care. DWEAC installed greater compassion and empathy among learners whilst acknowledging how workforce challenges can contribute to carer stress and emotional exhaustion [42]. Affective behaviours are not easily taught using traditional teaching methods, which suggests that the pedagogical methods in DWEAC are effectively triggering emotional reactions, most likely by offering a sense of how it feels to be “in the patient’s shoes” [43]. Despite support for emotive learning, DWEAC may be enhanced further from a greater focus on the emotional wellbeing of participants during training with additional strategies to safeguard wellbeing in practice [44].

Learning gains following DWEAC were not well established from quantitative findings. This is consistent with another quantitative study that used KIDE, CODE, and DAS as measures and suggested that dementia education has limited impact on the knowledge, attitudes, and confidence of health care staff [21]. Qualitative findings provided more support for learning gains, which highlights the importance of mixed methods when evaluating dementia training programmes [45]. The mixed methods findings are consistent with substantive evidence from a comprehensive systematic review that suggested that dementia training can improve knowledge and attitudes among acute care staff [37]. Improvements in attitudes and confidence among DWEAC participants were well qualified, with learners reporting increased empathy and greater overall job satisfaction when working with people with dementia. This is consistent with Murray et al. [46], who demonstrated increased levels of job satisfaction and confidence among clinical staff and ACSS following dementia training.

Measuring the extent to which learners change their on-the-job behaviours due to training can prove more difficult, since time is required for behavioural change to take place [33]. Dementia care scenarios provided interview participants with opportunities to contemplate behaviours with pre- and post-training questions after the programme had concluded. This approach has been used previously to evaluate the impact of dementia training among ACSS in the acute hospital setting [47]. Analysis of pre- and post-DWEAC behaviours demonstrated that participants had enhanced communication skills, person- and relationship-centred care practices, and awareness of ways to respond to patient behaviours.

The development of communities of practice and use of train-the-trainer programmes is known to support the wider dissemination of dementia knowledge in acute hospital settings [48]. DWEAC supported ACSS to develop and deliver training resources for other staff members and embed learning more extensively in practice [37]. This willingness to impart new knowledge is likely to have a profound impact on the acute care experience for people with dementia whilst simultaneously contributing to the sustainability of DWEAC [49].

## 5. Limitations

This pilot study aimed to understand the potential of DWEAC as an educational intervention for ACSS and provided insights into the feasibility of a wider-scale evaluation. ACSS had important expertise to contribute; however, competing demands on their time and the need to prioritise patient care may have resulted in engagement barriers, impacting the quantitative sample size and subsequently limiting the generalisability of the findings, and the findings are restricted to one health board in Scotland. There was also a risk of selection bias since baseline participant characteristics of those who did and did not attend DWEAC were not compared for similarities/differences. Furthermore, the measures used in the quantitative strand were not validated in the context of this evaluation and may not have accurately reflected the instructional content in DWEAC. Several DWEAC participants expressed an interest in being interviewed following the training; however, few consented, with subsequent difficulty achieving data saturation and limited transferability of the qualitative findings. This “unwillingness” to participate may have introduced a degree of volunteer bias impacting the reliability and validity of the evaluation [50]. A better understanding of factors that motivate ACSS engagement with research would be helpful in future evaluations [51]. The successful implementation of effective dementia education in the acute hospital is often dependent upon robust research evidence, which will require an organisational commitment with management support to prioritise dementia education and research, including support for staff who will implement the learning in practice [52].

## 6. Conclusions

This evaluation suggests that ACSS in this health board have good levels of dementia care competence and confidence despite access limitations to comprehensive dementia training programmes. DWEAC presents an opportunity for ACSS to learn about the dementia trajectory using reflective and interactive pedagogical practices that can effectively consolidate experiential learning towards a more person-centred and compassionate workforce. DWEAC may be strengthened by providing even greater support for emotional learning and the challenges encountered by ACSS in practice. Wider dissemination of the training will be dependent upon future evaluations using mixed or other design typologies that can achieve a nuanced understanding of staff learning needs and training impact with organisational support to motivate and enable staff engagement with dementia research and education.

### Implications for Environmental and Public Health Practice

There is growing evidence that the proportion of staff receiving dementia care training is low even in specialist units [53]. Dementia was declared as a public health priority, with workforce education identified as a key domain [54]. Dementia inclusive and enabling environments with a knowledgeable and skilled workforce are crucial to mitigate stigma and discrimination [55]. This can be best achieved by raising awareness through targeted staff education and training to make hospital environments dementia inclusive. Dementia education interventions such as DWEAC are public health responses for capacity building and enabling hospitals to become dementia inclusive.

## Figures and Tables

**Table 1:** Themed content in DWEAC.

Classroom Sessions				Associated Workbooks
Day	Themes	Duration	Facilitator	
1	Introduction to DWEAC and Workbooks	30 min	1, 2	
	The Human Brain	1 h	1	Dementia Care Essentials
	Memory & Sensory Changes in Dementia	1 h	1	
	Insights on Living with Dementia	30 min	1, 2	
	Person-centred Dementia Care	30 min	1, 2	
	Living with Dementia	45 min	3	
	Family Carers’ Perspectives	45 min	3	
2	Stages of the Dementia Journey	1 h	1, 2	Dementia Care Priorities
	Dementia, Delirium, and Depression	45 min	1, 2	
	Stress & Distress in Dementia	45 min	1	
	Dementia Inclusive and Enabling Environments	1 h	1	
3	Advanced Dementia ^a^	45 min	1, 2	Dementia Care Enablers
	Relationship-centred Dementia Care ^b^	45 min	1	
	Commitments & Pledges for Dementia Care	1 h	1, 2	

a Included end-of-life care and pain management. b Included staff wellbeing facilitator: 1 [nurse lecturer]; 2 [dementia nurse consultant]; 3 [expert/s with lived experience of dementia].

**Table 2:** Participant demographics [online survey].

Age	*n* [%]	Years of Acute Care Experience	*n* [%]
16–20	1 [3.3]	1–5 years	14 [46.7]
21–30	5 [16.7]	5–10 years	7 [23.3]
31–40	5 [16.7]	10–20 years	5 [16.7]
41–50	12 [40.0]	More than 20 years	3 [10.0]
51–60	3 [10.0]	Missing	1 [3.3]
>60	4 [13.3]		
Department	*n* [%]	Years of dementia care experience	*n* [%]
General Medicine	11 [36.7]	1–5 years	12 [40.0]
Medicine of the Elderly	11 [36.7]	5–10 years	5 [16.7]
Department of Clinical Neuroscience	3 [10.0]	10–20 years	8 [26.7]
Orthopaedics	3 [10.0]	More than 20 years	5 [16.7]
Stroke Medicine	2 [6.7]		
Role	*n* [%]	Frequency of dementia care	*n* [%]
Clinical Support Worker	14 [46.7]	On a daily basis	12 [40.0]
Healthcare Assistant/Care Assistant	5 [16.7]	At least on a weekly basis	13 [43.3]
OT Assistant	3 [10.0]	At least on a monthly basis	4 [13.3]
PT Assistant	2 [6.7]	Missing	1 [3.3]
Other	6 [20.0]		
Experience of informal dementia care	*n* [%]	Attended DWEAC training	*n* [%]
Yes	5 [16.7]	Yes	4 [13.3]
No	23 [76.7]	No	26 [86.7] *
Missing	2 [6.7]		

* Included 1 × missing response.

**Table 3:** Dementia knowledge.

	All Participants		Did Not Attend DWEAC		Attended DWEAC	
	Response	Correct *n* [%]	Response *n*	Correct *n* [%]	Response	Correct*n n* [%]
1. Anger and hostility occur in dementia mostly because the “aggression” part of the brain has been affected	30	18 [60]	26	16 [62]	4	2 [50]
2. Dementia is a general term which refers to a number of different diseases	30	23 [77]	26	20 [77]	4	3 [75]
3. Dementia can be caused by a number of small strokes	30	18 [60]	26	17 [65]	4	1 [25]
4. People with dementia will eventually lose all their ability to communicate	30	17 [57]	26	14 [54]	4	3 [75]
5. A person with dementia’s history and background plays a significant part in their behaviour	28	23 [82]	24	20 [83]	4	3 [75]
6. A person with dementia is less likely to receive pain relief than a person without dementia when they are in hospital	30	23 [77]	26	19 [73]	4	4 [100]
7. People with dementia who are verbally aggressive nearly always become physically aggressive	30	23 [77]	26	20 [77]	4	3 [75]
8. When people with dementia walk around it is usually aimless	29	22 [76]	25	19 [76]	4	3 [75]
9. Permanent changes to the brain occur in most types of dementia	30	29 [97]	26	25 [96]	4	4 [100]
10. Brain damage is the only factor that is responsible for the way people with dementia behave	30	28 [93]	26	24 [92]	4	4 [100]
11. Physical pain may result in a person with dementia becoming aggressive or withdrawn	30	29 [97]	26	26 [100]	4	3 [75]
12. People who have dementia will usually show the same symptoms	30	25 [83]	26	22 [85]	4	3 [75]
13. Currently, most types of dementia cannot be cured	30	30 [100]	26	26 [100]	4	4 [100]
14. People with dementia never get depressed	30	29 [97]	26	25 [96]	4	4 [100]
15. My perception of reality may be different from that of a person with dementia	29	29 [100]	25	25 [100]	4	4 [100]
16. It is possible to catch dementia from other people	30	28 [93]	26	24 [92]	4	4 [100]

**Table 4:** Dementia care confidence among ACSS who did and did not attend DWEAC.

	Did Not Attend [*n* = 26]	Attended [*n* = 4]
	Median [IQR]	Median [IQR]
*I feel able to …*		
Identify when a person may have a dementia	3 [2,4]	4 [3.5,4]
Understand the needs of a person with dementia when they cannot communicate well verbally	3 [2,4]	4 [4,4]
Manage situations when a person with dementia becomes agitated	3 [2.25,4]	4 [3.75,4]
Interact with a person with dementia when they cannot communicate well verbally	4 [2.25,4]	4 [4,4]
Work with people who have a diagnosis of dementia	4 [3.25,4.75]	4 [4,4]
Help a person with dementia feel safe during their stay in hospital	4 [3,4]	3.5 [3,4]
Gather relevant information to understand the needs of a person with dementia	4 [3,4]	4 [3.5,4]
Interact with a person with dementia when they can communicate well verbally	4 [3,5]	4 [4,4.25]
Understand the needs of a person with dementia when they can communicate well verbally	4 [3,5]	4 [4,4.25]
Total	4 [3,4]	4 [4,4]

1 = Not at all confident; 2 = Slightly confident; 3 = Somewhat confident; 4 = Quite confident; 5 = Extremely confident.

**Table 5:** Dementia attitudes among ACSS who did and did not attend DWEAC.

	Did Not Attend [*n* = 26]	Attended [*n* = 4]
	Median [IQR]	Median [IQR]
Positive items		
I feel confident being around people with dementia	5 [4,6]	7 [6.75,7]
It is rewarding to work with people with dementia	6 [4.25,7]	6.5 [6,7]
People with dementia can be creative	6 [6,7]	7 [7,7]
I am comfortable providing personal care to people with dementia	6 [4.25,6.75]	7 [7,7]
I feel relaxed around people with dementia	6 [4,6.75]	7 [6.75,7]
People with dementia can enjoy life	6 [6,7]	7 [7,7]
People with dementia can feel when others are kind to them	6 [6,7]	7 [7,7]
I admire the coping skills of people with dementia and their families	6.5 [6,7]	7 [7,7]
We can do a lot now to improve the lives of people with dementia and their families	6.5 [5,7]	7 [6.75,7]
Every person with dementia is unique and has different needs	7 [7,7]	7 [7,7]
People with dementia like having familiar things nearby	7 [6,7]	7 [7,7]
It is important to know the past history of someone with dementia	7 [6,7]	7 [7,7]
It is possible to enjoy interacting with people with dementia	7 [6,7]	7 [7,7]
Difficult behaviours may be a form of communication for people with dementia	7 [6,7]	7 [6.75,7]
Negative items		
I feel frustrated because I do not know how to help people with dementia and their families	3 [3,5.75]	5 [3.5,6.25]
I feel afraid being around people with dementia	6 [4,6]	6 [6,6.25]
I cannot imagine taking care of someone with dementia	6 [4,6]	6.5 [6,7]
I would avoid an agitated person with dementia	6 [4,6]	7 [7,7]
I feel uncomfortable being around people with dementia	6 [4,6]	7 [6.75,7]
I am not very familiar with dementia	6 [5,6]	7 [6.75,7]
All items	6 [6,6.625]	7 [7,7]

Positive items: 1 = Strongly disagree; 2 = Disagree; 3 = Slightly disagree; 4 = Neither agree nor disagree; 5 = Slightly agree; 6 = Agree; 7 = Strongly agree. Negative items: 1 = Strongly agree; 2 = Agree; 3 = Slightly agree; 4 = Neither agree nor disagree; 5 = Slightly disagree; 6 = Disagree; 7 = Strongly disagree.

**Table 6:** Participant quotes and interpretation.

Quote	Interpretation
*“When the pandemic hit … I wasn’t aware as of as many people, but now … if you went round every ward, they’ve all got, you know, quite a healthy number of elderly people with dementia”. P2.*	Interview participants described an increasing prevalence of patients with dementia in acute care wards, which were considered similar to MOE wards in terms of patient demographics, with this trend more apparent since the COVID-19 pandemic.
*“The beauty of the clinical support worker is they’re afforded the time more to talk to the patient because they’re making the bed, they’re doing the personal care … you help them, you sit them down, you do all the things, but you learn an awful lot about them when you’re able to talk to them”. P2.*	ACSS were well placed to provide good-quality dementia care given their unique opportunities to get to know patients well.
*“The holistic side wasn’t there, and not because nurses and doctors don’t want to do that, they just are so driven by time and pressure … we’re bursting at the seams with people coming in the hospital”. P2.*	The participants often strived to provide good-quality dementia care but were often constrained by busy workloads and time demands in practice.
*“Some people are just what I would call textbook nurses … they’ve gave them their medicine … they’ve gave them their fluids … they’ve gave them a basin … to me are not going the extra mile they need to go to find out what it is actually going on because, again, they’re, for me, removed from the dementia side”. P2.*	A significant barrier was the task-orientated nature of the acute care environment.

**Table 7:** Participant Satisfaction.

*“I don’t think I thought I’d meet so many people, get so much, you know, true life representation of dementia, I thought it was more sort of lecture based if you know what I mean”. P1*
*“I found it really, really insightful … because of the depth it went into”. P3.*
*“I think that was really useful … we had the time … after the first day, right, you go away and you think well there’s questions that I’ll ask the second”. P3.* *“Having the workbooks to fall back on, or read before, you know, before coming … and we’ve obviously got that as a reference now as well”. P3.*
*“I think it was good the way it was broken down into the different sections and it made you think about things a bit differently rather than seeing it holistically”. P3.*
*“I like things in front of me … I would have liked a sort of workbook so that I could highlight in … something printed”. P1.*
*“It’s great getting information, you know, online … but it’s good to be able to say to somebody, well, you know, hang on a minute I don’t agree with that … everybody’s got a different skills level, everybody got a different, you know, take on it, or experience”. P3.*
*“To know that if I’ve got any concerns … I would like to be able to think that I could come to you and say well how do we deal with this?”. P2.*
*“Listening to their story and, you know, having them standing in front of you, it wasn’t a piece of paper you were reading … they were standing in front of you … it gave you a complete insight as to day-to-day life actually living, and actually, it was quite positive, they were very positive, it wasn’t all negative” P1.*
*“I felt annoyed with myself because, you know, here’s these two wonderful people standing at the front, you know, sharing their story with us and I’m bubbling in the background”. P1.* *“They were bringing a tear to your eye … just because of their stories”. P3.*

**Table 8:** Learning gains following DWEAC.

Theme	Code	Quote
Knowledge	Dementia subtypes	“I didnae realise there was so many different types of dementia, I mean I knew some of them cause there’s a kind of common ones right, but I didnae realise that there was, you know, there was so many”. P3.
	Person-centred care	The course did get me thinking a lot more about the person-centred … it just kind of came to the forefront … given me a better understanding of what the person-centred approach was going to be”. P3.
	Relationship-centred care	“The information you’re going to find out from their families could be key”. P1.
	Informal dementia care	“I think it’s Lewy bodies that my mum’s got … it gave me a wee bit more understanding … it’s helped me professionally and yeah in my personal life”. P3.
	General	“Oh, it’s like night and day … I feel like I have a much better understanding now”. P1.
Skills	Communication	“It’s trying to listen to people and take on board what their saying rather than just your perception”. P3.
	General	“I feel much more equipped to be able to do my job and care for them the best way I can”. P1.
Confidence	General	“I’m less scared when I go in for a shift and I look at the handover sheet and it says that somebody’s got dementia … not scared for myself but scared in the fact that I’m not going to do the best by that patient”. P1.
Attitudes	General	“Hopefully my attitude and my dealing with people has changed for the better… I do have a bit more understanding, a bit more empathy for them”. P3.

**Table 9:** Dementia care scenario [Participant 1].

Mary is an 86-year-old lady living on her own with moderate dementia. She has been admitted to an MOE ward following a fall at home. She has bruises and pain but is deemed medically fit and is waiting for a care package before discharge. Staff have noticed a change in Mary’s presentation over the past 24 h. She is much more confused and believes that someone in the ward is trying to hurt her. What would be your assessment of Mary’s situation?	BEFORE DWEAC *“Before the training you would have just maybe just put it down to her, you know, dementia, and not looked at the bigger picture”*
	AFTER DWEAC *“I would have thought, kind of put the dementia to one side and … maybe took a urine sample and seen if she had a urine infection or delirium maybe”*

**Table 10:** Dementia care scenario [Participant 2].

On a visit to my dad Charlie, he was particularly agitated, ill at ease with himself and everyone around him. His language was ‘industrial’ at best. Staff thought that he was being aggressive but chose to ignore his agitation rather than try to get to the cause. Charlie was totally deaf in one ear and had a hearing aid in his other ear. We were asked whether it would be ok to increase his medication to sedate him more. What do you think is happening for Charlie, and how would you manage this situation?	BEFORE DWEAC *“I would be just going with my instinct with someone like that before this training course”*
	AFTER DWEAC *“If they canny walk properly there’s a reason … if they canny hear there’s a reason … if their eyesight’s bad and they’ve not got their glasses, where’s their glasses? If they’ve not got their teeth, how can they eat their food? So, all those things became more evident”*

**Table 11:** Dementia care scenario [Participant 3].

Elsie is 82 years old and usually lives at home on her own following the death of her husband five years ago. She was diagnosed with moderate dementia three years ago and has a package-of-care three times a day. Her two daughters and three grandchildren visit regularly and help with appointments and shopping. Elsie was admitted to hospital five weeks ago after developing sepsis secondary to an untreated urinary tract infection and has started to recover well. There have been incidents over the past five days where Elsie has been found banging on the ward door and trying to leave. Elsie believes that she needs to get home to collect her children from school. Attempts to reorientate Elsie have been unsuccessful and have resulted in her becoming very distressed and mistrusting of the ward staff. What do you think is happening for Elsie, and how would you manage this situation?	BEFORE DWAC *“I might have just, you know, tried to … appease her, and just, you know, ‘right come on Elsie’, you know, ‘you need to come back to your room’, or … ‘come on get a cup of tea’ … trying to calm her down but just, you know, well yes calm her down … just trying to get her to settle down”*
	AFTER DWEAC *“If I didnae already know what her family background was, trying to get her family story from her as much as you could”*

## Data Availability

The original contributions presented in this study are included in the article/supplementary material. Further inquiries can be directed to the corresponding author(s).

## Supplementary Materials

The following supporting information can be downloaded at https://www.mdpi.com/article/10.3390/ijerph22060860/s1.
