# Supplementary material for: Dementia Education and Training for In-Patient Health Care Support Workers in Acute Care Contexts: A Mixed-Methods Pilot Evaluation
Source: Int J Environ Res Public Health. 2025 May 30;22(6):860. doi: 10.3390/ijerph22060860 (PMC12192945; doi:10.3390/ijerph22060860)
Supplement: Supplementary file 1 [file ijerph-22-00860-s001.zip › Supplementary File S4_DWEAC_Interview Guide.pdf]

Project Title: Dementia Workforce Excellence in Acute Care [DWEAC]: A Mixed Method Study

Interview Topic Guide

Introduction

Thank you for agreeing to take part in this research. We are interested in understanding your experience of participating in Dementia Acute Care Workforce Capacity Building for Dementia Care Excellence. We expect the interview to last between 45 and 60 mins, but we can stop to take a break at any point, and you can finish the interview at any time without giving an explanation.

. May I double-check that you are happy for this interview to be recorded?

. [If participant has consented to be recorded, switch on the recorder]

. Establish identity and verbal consent for participation in interview.

Questions

1. Please could you share what motivated you to participate in the dementia training?
2. What were your expectations of the training and taking part?
3. Have you accessed any dementia training prior to this? If so, how did this training compare?
4. What was your understanding, experience and expectations of person-centered dementia care before this training?
5. How has your understanding, experience and expectations of person-centered dementia care changed since taking part?
6. How has this training informed or influenced your clinical practice?
7. Please read the following dementia care scenario[1]

- Please describe how you may have approached/ responded to this scenario before the training.

- Please describe how you might approach/ respond to this scenario since completing the training.

8. How have you applied what you learned in the training in your everyday practice?

9. What are the top three things you took away from your training?

10. What are your thoughts on the blended approach to dementia education (e.g., blending learning from asynchronous workbooks and live synchronous [face to face] sessions)?

11. What are your thoughts on the teaching and learning methods used in the training (e.g., access to experts/ case-based learning)

12. What would you have liked to have experienced more of in the training?

13. What would you have liked to have experienced less of in the training?

14. Would you be interested in delivering this training to your colleagues?

15. How would you design / deliver this training if had the opportunity?

16. Any other suggestions or thoughts including the duration, content, delivery, mode etc for future delivery of this training programme within NHS Lothian and across Scotland?

[1]Example of dementia care scenario

On a visit to my dad Charlie in an acute care ward he was particularly agitated, ill at ease with himself and everyone around him. His language might have been described as 'industrial' at best. Staff thought he was being aggressive but chose to ignore his agitation rather than try to get to the cause. We were asked whether it would be ok to increase his medication to sedate him more.

Charlie was totally deaf in one ear and had a hearing aid in his other ear. No one had thought to check his hearing aid which had no battery in it, not just a flat battery, no

battery at all. Charlie couldn't hear what was being said or asked of him, couldn't make himself understood and had become increasingly frustrated.

Once a new battery had been installed and hearing returned the agitation abated without the need for sedation. A simple check that caused incredible upset for Charlie and could have resulted in him being heavily sedated unnecessarily.
