# Supplementary material for: Dementia Education and Training for In-Patient Health Care Support Workers in Acute Care Contexts: A Mixed-Methods Pilot Evaluation
Source: Int J Environ Res Public Health. 2025 May 30;22(6):860. doi: 10.3390/ijerph22060860 (PMC12192945; doi:10.3390/ijerph22060860)
Supplement: Supplementary file 1 [file ijerph-22-00860-s001.zip › Supplementary File S1_Mixed Methods Interpretation.pdf]

SUPPLEMENTARY FILE S1:. MIXED METHODS INTERPRETATION

TABLE S1. THE NEED FOR DEMENTIA EDUCATION

		Quantitative findings			
		Not all ACSS feel prepared to care well for people with dementia	Many ACSS learn about dementia from work experience	Some ACSS have personal experience of informal dementia care	E-learning is the most common form of formal dementia training
Qualitative findings	There is an increasing prevalence of patients with dementia with acute care settings		e		
	Lengthy hospital stays are common for people with dementia in acute care		e		
	Staff workload and time pressures impact dementia care quality in acute care		d		
	Task orientation is a significant barrier to dementia care in acute settings		d		
	ACSS require knowledge and skills care well for people with dementia/family members	e			
	ACSS require knowledge and skills to support informal dementia care for family members	e		e	
	ACSS acquire skills for dementia care informally on the job	e	c		
	ACSS are well placed to provide good quality dementia		e		
	ACSS are unaware of opportunities for dementia education at work				e

ACSS [Acute care support staff]; c [convergent]; d [divergent]; e [expansion].
