# Supplementary material for: Dementia Education and Training for In-Patient Health Care Support Workers in Acute Care Contexts: A Mixed-Methods Pilot Evaluation
Source: Int J Environ Res Public Health. 2025 May 30;22(6):860. doi: 10.3390/ijerph22060860 (PMC12192945; doi:10.3390/ijerph22060860)
Supplement: Supplementary file 1 [file ijerph-22-00860-s001.zip › Supplementary File S2_Dementia Care Confidence and Competence Before DWEAC.pdf]

SUPPLEMENTARY FILE S2: DEMENTIA CARE CONFIDENCE AND COMPETENCE BEFORE DWEAC

		Quantitative findings		
		ACSS have high levels of dementia knowledge	ACSS have good levels of dementia care confidence	ACSS have positive dementia care attitudes
Qualitative findings	DWEAC participants demonstrated awareness of person- and relationship-centred care	e		
	DWEAC participants could make assumptions about people with dementia			d
	DWEAC participants had awareness of relevant dementia care policy and procedures	e		
	DWEAC participants demonstrated awareness of unmet needs in people with dementia	e		
	DWEAC participants cared for people with dementia based on instinct rather than robust knowledge	d		
	DWEAC participants could lack confidence to implement psychosocial interventions autonomously		d	

ACSS [Acute care support staff]; d [divergent]; DWEAC [Dementia education for workforce excellence]; e [expansion].
