# Supplementary material for: Dementia Education and Training for In-Patient Health Care Support Workers in Acute Care Contexts: A Mixed-Methods Pilot Evaluation
Source: Int J Environ Res Public Health. 2025 May 30;22(6):860. doi: 10.3390/ijerph22060860 (PMC12192945; doi:10.3390/ijerph22060860)
Supplement: Supplementary file 1 [file ijerph-22-00860-s001.zip › Supplementary File S3_Dementia Care Confidence and Competence After DWEAC.pdf]

SUPPLEMENTARY FILE S3_ DEMENTIA CARE CONFIDENCE AND COMPETENCE AFTER DWEAC

		Quantitative findings		
		DWEAC may have addressed some dementia knowledge gaps among ACSS	DWEAC may have resulted in improved dementia care confidence among ACSS	DWEAC helped improve dementia care attitudes among ACSS
Qualitative findings	DWEAC participants gained knowledge about common forms of dementia	e		
	DWEAC participants gained knowledge about person-centred care	e		
	DWEAC participants gained knowledge about relationship-centred care	e		
	DWEAC participants gained knowledge for informal dementia care	e		
	DWEAC participants developed a broader understanding about dementia	e		
	DWEAC participants developed awareness of physical, emotional, and unmet needs of people with dementia	e		
	DWEAC participants developed proficiency in their job and ability to care for people with dementia	e		
	DWEAC participants developed enhanced communication skills when working with people with dementia	e		
	DWEAC participants were more attuned to alternative pathologies when interpreting behaviours	e		
	DWEAC participants developed in confidence to share new knowledge with colleagues		e	
	DWEAC participants developed in confidence to work with people with dementia		c	
	DWEAC participants demonstrated greater attention to personhood			e
	DWEAC participants demonstrated greater care compassion and empathy			e

ACSS [Acute care support staff]; c [convergent]; DWEAC [Dementia education for workforce excellence]; e [expansion].
